# ADDRESSING THE NEEDS OF INCARCERATED INMATES WITH DEMENTIA: CREATING DEMENTIA-FRIENDLY PRISONS

**DOI:** 10.1093/geroni/igz038.1620

**Published:** 2019-11-08

**Authors:** Valerie Gruss, Memoona Hasnain

**Affiliations:** University of Illinois at Chicago, Chicago, Illinois, United States

## Abstract

U.S. prisons are experiencing a graying of their population, with many older inmates experiencing chronic conditions, including dementia. Older prisoners now represent 10% of the U.S. prison population and 18% of Illinois’ prison population. Aging inmates cost more to incarcerate due to their medical needs. Bureau of Prisons data estimate $881 million (19%) of its budget was spent to incarcerate aging inmates. Prisons are seeking solutions to address the unmet needs of older inmates, especially those with dementia. These older inmates with dementia face discrimination and exploitation within the prison population, and Correction Officers and clinicians lack training to understand and address their complex needs. Utilizing the Alzheimer’s Association’s, ACT on Alzheimer’s Toolkit we implemented four phases guiding communities’ adoption of dementia-friendly practices: we convened meetings with Illinois Department of Correction leaders, assessed community strengths and gaps by surveying prison wardens, analyzed findings and created an action plan to provide dementia training of prison staff to create a dementia friendly community. Our “Dementia-Friendly Prisons” program trains prison staff on understanding and providing supportive care and management to inmates with dementia, enabling staff to meet inmates’ needs, thereby creating an environment where inmates with dementia are safe and treated respectfully. Our program used an Appreciative Inquiry Four-D cycle approach (Discover-Dream-Design-Destiny) to engage and empower learners from a highly diverse workforce to develop into collaborative teams. Our program remedies a problem in the delivery of prison healthcare, serving a particularly vulnerable population and creates an adaptable model for other prisons and communities.

